# The Role of the Canine Gut Microbiome and Metabolome in Health and Gastrointestinal Disease

**DOI:** 10.3389/fvets.2019.00498

**Published:** 2020-01-14

**Authors:** Rachel Pilla, Jan S. Suchodolski

**Affiliations:** Gastrointestinal Laboratory, Department of Small Animal Clinical Sciences, Texas A&M University, College Station, TX, United States

**Keywords:** gut microbiome, gut metabolome, dog, gastrointestinal, probiotics, diarrhea, diet

## Abstract

The gut microbiome contributes to host metabolism, protects against pathogens, educates the immune system, and, through these basic functions, affects directly or indirectly most physiologic functions of its host. Molecular techniques have allowed us to expand our knowledge by unveiling a wide range of unculturable bacteria that were previously unknown. Most bacterial sequences identified in the canine gastrointestinal (GI) tract fall into five phyla: Firmicutes, Fusobacteria, Bacteroidetes, Proteobacteria, and Actinobacteria. While there are variations in the microbiome composition along the GI tract, most clinical studies concentrate on fecal microbiota. Age, diet, and many other environmental factors may play a significant role in the maintenance of a healthy microbiome, however, the alterations they cause pale in comparison with the alterations found in diseased animals. GI dysfunctions are the most obvious association with gut dysbiosis. In dogs, intestinal inflammation, whether chronic or acute, is associated with significant differences in the composition of the intestinal microbiota. Gut dysbiosis happens when such alterations result in functional changes in the microbial transcriptome, proteome, or metabolome. Commonly affected metabolites include short-chain fatty acids, and amino acids, including tryptophan and its catabolites. A recently developed PCR-based algorithm termed “Dysbiosis Index” is a tool that allows veterinarians to quantify gut dysbiosis and can be used to monitor disease progression and response to treatment. Alterations or imbalances in the microbiota affect immune function, and strategies to manipulate the gut microbiome may be useful for GI related diseases. Antibiotic usage induces a rapid and significant drop in taxonomic richness, diversity, and evenness. For that reason, a renewed interest has been put on probiotics, prebiotics, and fecal microbiota transplantation (FMT). Although probiotics are typically unable to colonize the gut, the metabolites they produce during their transit through the GI tract can ameliorate clinical signs and modify microbiome composition. Another interesting development is FMT, which may be a promising tool to aid recovery from dysbiosis, but further studies are needed to evaluate its potential and limitations.

## Introduction

The gut microbiome is composed of bacteria, archaea, viruses, and eukaryotic organisms that reside in the gastrointestinal tract, and which relate with the host in a symbiotic fashion. For example, bacteria in the guts produce short-chain fatty acids (SCFA) that nourish the intestinal epithelium, while the epithelium produces mucus that feeds beneficial bacteria.

The gut microbiome contributes with metabolic functions, protects against pathogens, educates the immune system, and, through these basic functions, affects directly or indirectly most of our physiologic functions. Serotonin, a neurotransmitter, is mostly produced in the intestine, which has led to the development of the gut-brain axis concept (1). A healthy and stable microbiome can simultaneously act as pro- and anti-inflammatory, keeping a balance to prevent excessive inflammation while still being able to promptly respond to infections (2).

## Healthy Dog Microbiome

### Variations Along the Gastrointestinal GI Tract

Studies using bacterial culture or molecular methods are in agreement, demonstrating that abundance and richness of bacteria increase along the tract (3). Initial studies with bacteriological culture reported bacterial loads in the small intestine of healthy dogs to be lower than the colon, with overall load ranging along the gastrointestinal tract from 10^2^ to 10^11^ colony forming units (CFU) per gram of luminal content (4, 5). Molecular methods have allowed the identification of non-culturable bacteria present within the canine GI tract, and estimates of the total microbial load now range between 10^12^ and 10^14^, about 10 times the number of cells present in the host (6).

The microbial communities along the tract vary to reflect the microenvironment and physiological functions of each intestinal segment. For example, the small intestine harbors a mixture of aerobic and facultative anaerobic bacteria, while the colon is colonized almost exclusively by anaerobes. Along the GI tract, bacterial sequences typically belong to one of five phyla: Firmicutes, Fusobacteria, Bacteroidetes, Proteobacteria, and Actinobacteria (3, 7).

Differences in taxa abundance along the GI tract are reflected in the production and consumption of different metabolites. Along the tract, the concentration of most of the metabolites either increase or decrease progressively, although some will abruptly decrease at the end of the ileum, or sometimes even oscillate along the tract (7). Metabolomics, i.e., the study of metabolites, is a new field of research that attempts to capture and analyze the metabolic exchanges between host and microbiome. Metabolomics data can be considered complementary to metagenomics in the study of the gut microbiome, and allows scientists to move beyond the question “which microorganisms are there?” to the perhaps more pressing question “what are they doing?”

Despite the variations of taxa along the GI tract, samples from specific regions of the tract are difficult to obtain, and therefore most clinical studies focus on the fecal microbiota. Canine fecal samples reliably present most of the relevant taxa, unlike humans, in which most significant taxa are closely associated with the mucosa (8). Those findings could be related to the anatomy of the canine GI tract, shorter than that of humans, and with a faster transit time, and facilitate the study of the gut microbiome of dogs.

While variations in composition are observed between different studies, it is important however to note that regardless of the methods used, key bacterial species are consistently present in fecal samples of healthy dogs indicating the presence of a core fecal bacterial community. The fecal microbiome of healthy dogs is co-dominated by three phyla: Fusobacterium, Bacteroidetes, and Firmicutes (9, 10). When reviewing the literature, a wide variation in percentages of specific bacterial taxa can be seen. It is important to remember that the methods for sequencing and data analysis are in constant evolution, and much of those variations can be attributed to different sequencing and/or data analysis methods. Indeed, even different sequencing depths (i.e., the number of sequences per sample) can reduce the similarity of data, and new methods have significantly increased the numbers of sequences per sample that can be obtained. In addition, individual variations in the microbiome profile exist (11, 12) and should be taken into account especially when extrapolating findings from small sample groups.

Within this core bacterial community, many major taxa belong to phylum Firmicutes. Bacterial class Clostridia is consistently within the most abundant taxa, dominated by three *Clostridium* clusters: IV (e.g., family *Ruminococcaceae, Faecalibacterium prausnitzii*), XI (e.g., family *Peptostreptococcaceae*), and XIVa (e.g., family *Lachnospiraceae, Blautia* spp.) (8, 13, 14). Besides Clostridia, additional prevalent classes within the phylum Firmicutes are Bacilli and Erysipelotrichi. The class Bacilli consists almost exclusively of the order Lactobacillales, dominated by the genera *Streptococcus* and *Lactobacillus*. The class Erysipelotrichi mainly comprises the genera *Turicibacter, Catenibacterium*, and *Coprobacillus* (14, 15).

Bacteroidetes is another abundant phylum in fecal samples from dogs, comprising the genera *Prevotella, Bacteroides*, and *Megamonas* (10, 14). The most abundant genera, *Bacteroides* and *Prevotella*, were found to be highly variable in abundance between dogs. Interestingly, the combined *Prevotella* and *Bacteroides* abundances seem to be inversely related to phylum Fusobacteria abundance, which might indicate that they occupy the same niche (8).

Within phylum Fusobacteria, genus *Fusobacterium* is associated with healthy controls dogs. Interestingly, in humans *Fusobacterium* is associated with gastrointestinal disease, indicating *Fusobacterium* plays a different role in the GI tract of dogs (8). *Fusobacterium* abundance is increased in dogs with access to the outdoors (16), and higher levels of *Fusobacterium* are also seen in other carnivore species (17–19).

Phyla Proteobacteria and Actinobacteria are also commonly identified. These phyla are typically colonizers of the small intestine and in physiological conditions will present in smaller numbers in fecal samples. For example, members of the family *Enterobacteriaceae* (e.g., *Escherichia coli*) are facultative anaerobes, which allows them to take advantage of the oxygen available in the small intestine. In fecal samples their increase is associated with many diseases, as will be discussed further in this review. Actinobacteria are also associated with the small intestine, and include families *Corynebacteriaceae* (e.g., *Corynebacterium* spp.) and *Coriobacteriaceae* (e.g., *Collinsella* spp.) (7).

### The Effect of Diet

Dogs in their natural state are carnivorous scavengers, meaning that they thrive on a diet that is rich in meat, but will take advantage of any available food. In dogs, most microbiome studies have relied on extruded diets (also known as kibble), which represent up to 95% of the dry dog food market. Traditionally, the extrusion process requires a high load of carbohydrates, which is achieved with the inclusion of vegetable ingredients. However, alternative industrial processes have recently become available and a percentage of the pet food market now includes kibble with reduced carbohydrate content and increased protein content. Also increasingly popular are raw diets, frozen or freeze-dried, which are typically meat based and include low to negligible carbohydrate percentages.

Several studies in different species have shown that diet composition—especially large macronutrient differences like those found in carnivore vs. herbivore diets—is reflected in different gut microbiome profiles. In omnivore species, including humans, who can tolerate and thrive on either end of the spectrum, the short-term consumption of diets composed entirely of animal or plant products is enough to alter the microbial community structure and overwhelm inter-individual differences in microbial gene expression (20). In humans, the consumption of an animal-based diet increases the abundance of bile-tolerant microorganisms and decreases the levels of Firmicutes, which includes species known to metabolize dietary plant polysaccharides. In dogs, similar to humans, increases in vegetable fiber content in extruded diets leads to increases in the overall abundance of Firmicutes and decreases in Fusobacteria and Proteobacteria (9, 21).

However, for dogs, the kingdom of origin of the ingredients seems to be less important than the overall macronutrient composition. Extruded diets with similar macronutrient contents, but prepared exclusively with vegetable sources of protein, do not seem to significantly alter the microbiome of dogs when compared to traditional (mixed animal and vegetable) extruded diets (22).

A few studies have evaluated the impact of meat-based raw diets in the gut microbiome of healthy dogs in comparison with kibble-fed dogs. In one study (23), dogs were fed home-prepared *Bones and Raw Food* (BARF) diets consisting of a combination of raw meat, organs, meaty bones, and vegetables. Overall, compared to the kibble-fed control group, BARF diets included more protein and fat, and less fiber and carbohydrates. Another study (19) evaluated a red meat diet, containing exclusively bovine meat, organs, bones, and a mineral supplement to meet the guidelines from the Association of American Feed Control Officials (AAFCO). The red meat diet contained more protein, but less fat, fiber, and carbohydrates than the kibble control.

Both diets differed significantly in macronutrient content compared to commercial kibble diets, including less fiber and carbohydrate, and more protein, and resulted in similar microbial population shifts when compared with the kibble-fed control groups. In both studies, dogs fed raw diets had an overall decrease in the abundance of Firmicutes (23), including genera *Peptostreptococcus* and *Faecalibacterium*, and of genera *Bacteroides* and *Prevotella* (phylum Bacteroidetes) (19). Most of those genera are associated with digestion of dietary fiber and SCFA production, indicating a decrease in fermentation of fiber and carbohydrates due to their decreased intake. In contrast, other bacterial taxa were found to increase in abundance, including Proteobacteria and Fusobacteria (genus *Fusobacterium*), and two genera from phylum Firmicutes (*Lactobacillus* and *Clostridium*) (19, 23).

In those studies, dogs were fed the BARF diet for at least 4 weeks (4 weeks−9 years) (23), and the red meat diet for 3–9 weeks (19). One study with dogs receiving a raw diet for at least 1 year have found them to have a richer and more even microbiome compared to kibble-fed controls (24). They also showed an increased abundance of *Clostridium perfringens* and *Fusobacterium varium*, and a decreased abundance of *Coprobacillus* sp. compared to controls. However, the study (24) included only six animals, and studies with larger cohorts are needed to confirm those results.

In another study (25), healthy dogs were switched to a diet consisting of kibble mixed with increasing percentages of minced beef meat. Due to the lack of formulation to meet nutritional requirements, combined with the short observation period (only 1 week for each combination), results need to be interpreted with caution. Despite that, they reported similar results, with a decrease in *Faecalibacterium* and an increase in two *Clostridiaceae* strains.

Interestingly, one of the *Clostridiaceae* strains was later identified as *Clostridium hiranonis*, a bacterial species associated with normal bile acid (BA) metabolism (25, 26). A study (23) reported normal BA metabolism in healthy dogs fed BARF diets, with no significant difference from kibble-fed controls. BA metabolism is an important pathway not only for lipid digestion, but also for regulation of intestinal inflammation, and is commonly altered in chronic gastrointestinal diseases (26, 27).

Despite being commonly associated with gastrointestinal disease (due to *Clostridium perfringens* and *Clostridioides difficile*, potential pathogens which will be further discussed later in this manuscript), it has been suggested that increases in abundance of *Clostridiaceae* members (e.g., *Clostridium*) when protein-rich diets are fed to dogs may not be detrimental to their health (19), but rather associated with protein digestion. Increases in the family *Clostridiaceae* have been found to positively correlate with dietary protein content (19). In addition, *Clostridiaceae* were also found to positively correlate with protein digestibility and negatively correlate with fecal protein content (i.e., more *Clostridiaceae* results in less left-over protein in the feces). These findings suggest that *Clostridiaceae* may have a role in the metabolism of protein in the intestinal tract of dogs, different than the role played in the large bowel of the rat, where *Clostridiaceae* respond to dietary carbohydrates. In addition, *Clostridiaceae* had a positive correlation with fecal health score (i.e., feces were firmer) and a negative correlation with fecal output (i.e., less fecal output).

It is important to use caution when extrapolating findings from omnivore species to carnivores. The impact of diet in *Bifidobacterium* spp. (*Bifidobacteriaceae*), *Lactobacillus* spp. (*Lactobacillaceae*), and *Faecalibacterium* spp. (*Ruminococcaceae*) abundances is often investigated as they are considered beneficial in omnivores (28). Their benefit is attributed to their role in the production of carbohydrate fermentation products that are later converted into butyrate through the butyryl-CoA:acetate-CoA-transferase pathway. The role of butyrate, a SCFA, in intestinal health is undisputed, as butyrate is the preferred energy source for colonocytes (29).

However, butyrate can be found in fecal samples of all mammals regardless of their food sources. Therefore, in mammals that consume little to no carbohydrates, alternative pathways for butyrate production must be present. In a study with high-fat and low-starch diet (supplemented with lard) in dogs, acetate, butyrate and propionate levels were not different from dogs fed a low-fat high-starch diet (supplemented with maize and broken rice), indicating that the production of SCFA in dogs is not exclusively dependent on carbohydrate content (30). Supporting that hypothesis, another study (25) found that the addition of minced meat to a conventional kibble diet actually led to a small increase in fecal butyrate and isovalerate.

A recent study has highlighted that in carnivores, *Clostridiaceae*, and in particular *Clostridium perfringens*, are associated with the butyrate kinase butyrate-synthesis pathway, which allows the production of butyrate from protein (17). Another bacterium known to produce butyrate from protein sources is *Fusobacterium varium* (31), which was more abundant in a group of dogs fed meat-based raw diets for at least 1 year, suggesting an adaptation of the microbiome to the long-term diet (24). In addition, members of the *Fusobacteriaceae* family have been found to be more abundant in other carnivore species [cats: (18, 32), wolves: (33, 34), other carnivora: (17, 35)], and dogs fed raw diets (19, 23, 36).

Those findings bring into question whether bacteria that specialize in carbohydrate fermentation bring the same benefits described in omnivores to the carnivore GI tract (19). It is possible that in carnivores the butyrate production may be at least partially accomplished by other bacterial species such as members of the *Clostridiaceae* and *Fusobacteriaceae* families, which could be the reason for their increase in dogs fed raw diets.

BARF diets have also been found to increase fecal levels of gamma-aminobutyric acid (GABA), a neurotransmitter, and its precursor gamma-hydroxybutyric acid (GHB) (23). GABA and GHB are quickly absorbed from the GI tract when administered orally (37, 38), and foods rich in GABA-producing bacteria are available in Japan for the treatment of hypertension (39). The connection between the gut and the brain has been studied in many diseases, in dogs and other species, and has led to the development of the concept of the gut-brain axis (40).

Another neurotransmitter, serotonin, is essential for gut health. About 90% of the serotonin produced in the body originates from the intestines, where it regulates motility, secretion and blood flow through the enteric nervous system (41). Serotonin production is also partially controlled by the microbiome, either by direct production of serotonin by bacteria (42), or by consumption of its precursor, the amino acid tryptophan (1). The gut microbiota is essential for the development of the enteric nervous system. Germ-free mice display abnormally increased motor activity, and decreased anxiety responses, which normalize after colonization with microbiota from conventional mice (43).

### Establishment, Stability, and Decline of the Gut Microbiome

Regardless of the species, GI tract colonization in mammals starts even before the newborn exits the birth canal. The initial colonization varies and reflects the method of parturition and nutrition, and the establishing microbiome will increase in diversity over time (44). In humans, infants delivered vaginally acquire microbial populations from the mother's vaginal microbiota, while infants delivered through cesarean section acquire bacterial populations from their mother's skin (45). While no studies were performed with dogs born via cesarean section, newborn canines are exposed from birth to the dam's vaginal and fecal microbiota through the dam's tongue, and therefore the effect of the delivery method is likely less pronounced.

In dogs, similarly to humans, the maturation of the microbiome into an adult-like composition coincides with weaning. In a study with puppies aged 1 week to 1 year old (46), puppies had significantly different microbiomes during the first few weeks of life, with a predominance of Proteobacteria. At 9 weeks of age, however, Proteobacteria were significantly decreased, and *Faecalibacterium* spp. and *Clostridium hiranonis* were significantly increased, with values within the reference interval of healthy adults. In addition, adult littermates have been found to present a more similar microbiome composition than unrelated dogs, which hints to the importance of genetics and early life exposure (10).

The environment, and in particular other members of the household, can have an impact on the gut microbiome. In a study comparing dogs and their owners, significant sharing of skin microbiota between dog-owner pairs was seen when compared to other non-household members, and a smaller effect was seen also in fecal microbiota (16). While the overall impact of this microbiota sharing is likely small, it should be kept in mind in households that include immunocompromised individuals due to the potential zoonotic impact (47).

In many species, the gut microbiome is typically stable in healthy adults over time. In dogs, only the short-term variability has been evaluated, and the microbiome was found to be relatively stable over the period of 2 weeks (14). In a study of adult humans not taking antibiotics, more than 70% of fecal bacterial species within an individual were stable over 1 year, and calculations indicate that most species were likely stable over decades in weight-stable individuals (48). Although long-term data is not available for dogs, it is reasonable to expect that the gut microbiome might be stable in healthy adult animals, potentially all through their adult lives. A subset of bacterial taxa has been identified as keystone bacteria for gastrointestinal health (8), and used to create a Dysbiosis Index that can assess the gut microbiome through a set of qPCR reactions (49). The Dysbiosis Index will be discussed further later in this review.

Gastrointestinal microbial diversity was shown to decrease with age in other species, and that decline is associated with increased frailty and reduced cognitive function (50). Immunosenescence in elderly patients is associated with inflammaging, a chronic low degree inflammatory condition that includes imbalances in the microbiome composition (51). In a study with an exceptionally long-lived bat species, it was found that the microbiome from healthy old bats was very similar to that of juvenile bats, indicating a link between healthy aging and the gut microbiome (52). The aging dog microbiome hasn't been studied yet, and further research is needed to evaluate whether strategies to delay the loss of microbiome diversity in the elderly could also delay the onset of immunosenescence and increase longevity.

## Gut Microbiome in Disease

While age, diet, and environmental factors may play a significant role in the maintenance of a healthy microbiome, the alterations they cause pale in comparison with the alterations found in diseased animals. Many diseases, systemic or localized, impact or are impacted by the gut microbiome, and are associated with dysbiosis.

Gut dysbiosis is defined as alterations in the composition of the gut microbiota that result in functional changes in the microbial transcriptome, proteome, or metabolome (53). The increase in abundance of facultative anaerobic bacteria of the family *Enterobacteriaceae* is a common marker of dysbiosis (54), seen also in dogs (8).

It has been speculated that oxygen might be responsible for changes in the microbiota composition observed in dysbiosis (55). This hypothesis focuses on the availability of oxygen in the intestinal lumen, which can increase in situations that allow increased gut permeability, including inflammation (54). The resulting increase in free oxygen negatively impacts strict anaerobe populations, and driving an uncontrolled luminal expansion of facultative anaerobes, especially members of the *Enterobacteriaceae* family (53). The concept that oxygen, alone or in combination with other respiratory electron acceptors, controls the abundance of *Enterobacteriaceae* in the large bowel has important ramifications for understanding how a disruption in gut homeostasis drives dysbiosis.

The composition of the gut microbiota also has significant effects on immune function, and regulates the local production of antibodies. Although gut microbes are separated by the inner mucous layer and glycocalyx from direct contact with enterocytes, intestinal dendritic cells can extend their dendrites into the intestinal lumen and sample the microbiota. Most of these invading bacteria are killed by macrophages, and some are also presented to B cells. The B cells produce IgA, which is secreted into the lumen, binding to bacteria and activating targeted bacterial destruction (2).

Intestinal helper T (Th) cell precursors can differentiate into either Treg or Th17 cells depending on the signals received from the microbiota (2). In homeostasis the production of Treg cells is favored, that of Th17 cells is suppressed, and minimal inflammation occurs within the intestinal wall. In the absence of Treg cells, uncontrolled effector T cells will respond to microbial antigens and trigger inflammation (2). Specific bacterial groups can influence this process: as an example, members of the *Clostridium* groups IV and XIVa were shown to stimulate the induction of Treg (56), inducing an anti-inflammatory response, while segmented filamentous bacteria (SFB) were shown to induce Th17 (57), generating pro-inflammatory signals.

Intestinal inflammation can also be triggered by gut dysbiosis through bile acid dysmetabolism, which is seen both in dogs and in humans (26, 27, 58). Bile acids (BA) are essential for lipid digestion, but also play a role in mucosal defenses and have anti-inflammatory properties. Bacteria in the intestinal lumen are responsible for BA deconjugation and dehydroxylation, therefore dysbiosis can impair the production of secondary BA. Chronic intestinal disease can also decrease expression of the apical sodium-dependent bile acid transporter (ASBT), which is essential for the reabsorption of conjugated primary BA (27). Taken together, these results indicate that dysbiosis and intestinal inflammation can significantly impair BA metabolism, which in turn can further stimulate intestinal inflammation.

Dysbiosis is seen in many pathologies, both locally, within the gastrointestinal tract, and systemically (59). While outside of the scope of this review, recent work has associated dysbiosis with obesity (60), metabolic diseases (61), cancer (62), neurological disfunctions (63), and many others, both in dogs and in humans. However, caution should be taken when interpreting those findings. While an association with dysbiosis has been demonstrated in these diseases, often a causation effect is yet to be proven, and the dysbiosis may be a symptom of the disease process rather than its cause.

### Gut Microbiome and GI Diseases

Gastrointestinal dysfunctions are the most obvious association with gut dysbiosis. The gut microbiome has been found to be altered during both acute and chronic diarrhea. Like with healthy dogs, studies in dogs with GI diseases will report different taxa abundance percentages, however most taxa are consistently increased or decreased within the same disease phenotype.

Much of the apparent discrepancy between studies can be attributed to the difficulty obtaining samples from well-characterized clinical cases without confounding factors like recent antibiotic administration. This difficulty, paired with budget restrictions, results in studies with small numbers of samples, which limits statistical power. New technologies are making metagenome sequencing more accessible and, with increasing numbers of samples per project, such methodology issues should become easier to overcome.

In acute uncomplicated diarrhea (AD), dogs will develop a strong dysbiosis with a decrease in short-chain fatty acid (SCFA)-producing bacteria like *Blautia* spp., *Ruminococcus* spp., *Faecalibacterium praunitzii*, and *Turicibacter* spp. (64), and increased abundance in the genus *Clostridium* (26). Microbial diversity is decreased, and microbial communities differ significantly from healthy dogs.

Despite its mild clinical presentation, AD is associated with fecal dysbiosis that significantly alters not only fecal SCFA profiles but also blood and urine metabolites, suggesting that acute episodes of diarrhea have an impact on the overall metabolic profile of the host. In fact, a study (65) demonstrated that, while the abundance of SCFA-producing bacteria was decreased in fecal samples of dogs with AD, when SCFA were measured only propionate concentration was significantly decreased. Butyrate was instead found to be increased in the fecal samples of dogs with AD, a contradiction the authors suggest could arise from a decrease in butyrate absorption, or decreased utilization of butyrate by the enterocytes. Interestingly, though, they also demonstrated an increase in abundance of *Clostridium* sp., which as previously mentioned can produce butyrate from protein using an alternative pathway, which could be another explanation for the increase of butyrate.

Similar alterations have been detected in dogs with acute hemorrhagic diarrhea syndrome (AHDS), also known as hemorrhagic gastroenteritis (HGE) (66). Despite the difference in clinical presentation, dogs with AD and AHDS present similar shifts in bacterial groups (65). Compared to healthy dogs, both dogs with AD and AHDS have lower abundance of *Ruminococcaceae*, and *Faecalibacterium* spp. Studies have shown an association between *Clostridium perfringens* and AHDS (66), however, its enterotoxin could not be detected in AHDS fecal samples (67). The gene from the newly discovered netF toxin has been detected in the genome of *C. perfringens* isolated from intestinal biopsies of dogs with AHDS (68). In addition, other studies found a strong correlation between the presence of the netF gene in fecal samples and AHDS (69), and recovery from AHDS was accompanied by a significant decreased in netF gene and *C. perfringens* abundance (70). Combined, these results suggest that the netF toxin may play a role in the necrotizing lesions present in AHDS.

Another Clostridia that has gained much attention in human medicine, *Clostridioides difficile* (previously known as *Clostridium difficile*) (71) is a contradictory issue in dogs. While *C. difficile* infections in humans are well-studied and associated with antibiotic therapy and hospitalization, in dogs *C. difficile* and its toxins are detected in clinically healthy subjects, and infection cannot be induced in healthy dogs even after antibiotic therapy. In fact, one study (72) reported isolation rates of 29% in healthy volunteer dogs in Japan, and 35% in patients of a veterinary hospital in treatment for non-GI related conditions. However, other studies give more conservative isolation rates, with 5.5% of shelter dogs in Germany positive for *C. difficile* (73) and no isolates out of 55 healthy dogs in Canada (74).

*C. difficile* strains isolated from dogs are capable of producing toxins *in vitro* that severely impair tight junctions in canine and human cell lines (75). Authors have speculated that, similarly to humans, the presence of bile acid dehydroxylating bacteria, specifically *Clostridium hiranonis*, can be a protecting factor in dogs. In addition, *Sphingobacterium faecium* was also suggested as a protective species, which could be associated with its sphingophospholipid production abilities (75).

In symptomatic dogs found to be positive for *C. difficile*, it is unknown if clinical signs are attributable to *C. difficile*, or if it is a secondary finding. In an interesting study (76), in five dogs with chronic diarrhea that tested positive for *C. difficile*, diarrhea recurred after treatment with metronidazole, but ceased after diet intervention, and *C. difficile* was no longer detectable. These results suggesting that *C. difficile* was secondary to an underlying issue. Given the frequent identification of human epidemic PCR-ribotypes in dogs (72, 77), the potential of *C. difficile* as a zoonotic agent should be monitored (78).

The development of chronic enteropathies (CE) has been documented in dogs following episodes of parvovirus infection (79), and a similar pattern has been described in humans (80, 81). Some of the alterations present in acute diarrhea, both in humans and dogs, are also occurring in CE. Examples include dysbiosis and the decrease in SCFA producing bacteria, which has been found in dogs with acute and with chronic diarrhea (64, 65, 82). Further studies are needed to evaluate the long-term impact of acute diarrhea and its role in the development of CE.

Chronic enteropathies in dogs are generally classified according to their response to treatment as food-responsive diarrhea (FRD), antibiotic-responsive diarrhea (ARD), and immunosuppressant-responsive diarrhea (SRD, also known as idiopathic inflammatory bowel disease, IBD). All dogs with chronic enteropathies will present intestinal inflammation to some degree, and therefore share similarly dysbiotic microbiomes when compared to healthy dogs (83).

In addition to dysbiosis, dogs with CE also present significantly decreased fecal bacterial diversity (82, 84). In dogs with IBD, abundance of phylum Fusobacteria is reduced, along with phylum Bacteroidetes, especially families *Bacteroidaceae* and *Prevotellaceae* (e.g., genus *Prevotella*) (82, 83). Within the phylum Firmicutes, decreases in the families *Ruminococcaceae* (genus *Ruminococcus*), *Veillonellaceae* (genus *Megamonas*), and *Lachnospiraceae* were observed in dogs with IBD (82–84). Due to their role as SCFA-producing core bacteria, the simultaneous decrease in all these bacterial taxa reduces the availability of SCFAs, which are the main energy source for colonocytes (82). In addition, Gamma-Proteobacteria (e.g., *Enterobacteriaceae*), a hallmark of dysbiosis, are overrepresented in fecal samples of dogs with CE (8, 82, 85, 86).

When specific genera were assessed in fecal samples by qPCR, the abundances of *Blautia* spp. (Class Clostridia), *Faecalibacterium* spp. (Class Clostridia), and *Turicibacter* spp. (Class Erysipelotrichia) were significantly decreased (82, 84). In addition, *Fusobacterium* spp. (Class Fusobacteriia) and *Clostridium hiranonis* (Class Clostridia) were also decreased, and *Streptococcus* spp. (Class Bacilli) and *E. coli* (Class Gammaproteobacteria) were increased (82). Based on the specific knowledge accumulated from multiple molecular studies (8, 87), a series of qPCR reactions was developed to quantify gut dysbiosis in canine fecal samples. The mathematical model developed (49) uses the quantification of total bacteria and a panel of seven bacterial groups: *Faecalibacterium* spp., *Turicibacter* spp., *Escherichia coli, Streptococcus* spp., *Blautia* spp., *Fusobacterium* spp., and *Clostridium hiranonis* to calculate the Dysbiosis Index (DI). Negative DI values indicate normobiosis, and a positive DI values indicate dysbiosis. The DI is the first tool that allows quantification of gut dysbiosis, and can be used to monitor dysbiosis over time and in response to treatment. Other studies have since confirmed that DI is increased in dogs with CE (26, 27, 82).

In addition to SCFA, alterations in amino acids like tryptophan have also been found to significantly correlate with chronic enteropathies. Tryptophan is an essential amino acid in dogs, and a precursor for compounds such as kynurenine, serotonin, melatonin, and indole. The kynurenine pathway comprises at least 90% of tryptophan catabolism, and is rate-limited by the enzyme indoleamine 2,3, dioxygenase 1 (IDO-1). Humans with IBD have been found to have increased IDO-1 expression leading to lower serum tryptophan concentrations. Similar results have been seen in cats with CE, where serum tryptophan levels inversely correlate with disease severity (88). Increased tryptophan catabolism limits the production of serotonin, a neurotransmitter essential for GI secretion, motility and pain perception (89).

Tryptophan availability can also influence gut microbiota directly, as tryptophan is the precursor for the production of indole compounds. Indole compounds can only be synthesized by bacteria, and have been shown to increase expression of genes associated with improved gut homeostasis, decreased gut permeability, and increased mucin production in other species (90, 91). Tryptophan was the only amino acid found to be decreased in serum from dogs with protein-losing enteropathy, a form of chronic enteropathy, and lower serum tryptophan was correlated to lower serum albumin and poorer outcomes (92). In addition, in dogs with IBD, several indole compounds were found to be significantly decreased in fecal samples (93).

While dogs with FRD or IBD are not different in terms of global richness, diversity, or composition of the microbiota before treatment, their response to treatment does differ (94). After treatment, both dogs with FRD and dogs with IBD showed an increase in abundance of *Bacteroides*, which is associated with a healthy microbiome, in the colon. However, a few specific bacterial taxa presented different abundances between FRD and IBD. Dogs with FRD had a decrease of *Enterococcus* spp., *Corynebacterium* spp., and Proteobacteria, all potential pathogens, in the duodenum after treatment. In another study focusing on dogs with FRD (22), after an elimination dietary trial with a vegetable protein diet, the microbiome diversity was no longer significantly different from healthy controls, and richness was significantly increased.

Unlike FRD, however, in dogs with IBD treated with immunosuppressive therapy, with or without antibiotics or other therapeutic measures, clinical recovery is not always accompanied by a recovery of the microbiota. In one study (84), although all dogs recovered clinically, the diversity indices after 3 weeks of therapy showed a trend toward a further decrease. Another study evaluated recovery of bile acid metabolism and DI over 3 months, and, while BA metabolism was restored and *C. hiranonis* significantly increased, other relevant species and the overall DI were still significantly altered (26).

The difference in response to treatment between dogs with FRD and dogs with IBD can likely be attributed to the differences in the pathogenesis of the enteropathy. While dogs with IBD have an inflammatory process that seems to arise from a combination of genetic predisposition and environmental factors, dogs with FRD have an inflammatory process that is driven by the constant presence of an antigen of alimentary origin. Once the antigen is removed from the diet, the inflammation recedes allowing the microbiome to return to a state of normobiosis.

### Treatment Strategies and Their Impact on the Microbiome

Manipulations of the microbiome are often included as part of the treatment of GI diseases. Antibiotics, probiotics, and fecal transplants work by either eliminating detrimental bacteria, or by introducing beneficial bacteria. However, the manipulation of such a complex bacterial community is not simple, and often produces mixed results.

Antibiotics are used in acute as well as chronic gastrointestinal diseases, with the goal of removing pathogenic bacteria. However, antibiotics have serious consequences in the gut microbiota and often there is not enough evidence to justify their use. In dogs with AHDS, for example, a double-blind clinical trial in non-septic dogs found no difference in mortality rate, duration of hospitalization, severity of clinical signs, or outcome between the antibiotic and the placebo group (95). Chronic diarrhea is also often treated with antibiotics, however, a study (96) found no difference in clinical recovery in dog receiving metronidazole and prednisone vs. dogs receiving prednisone alone. Therefore, the appropriateness of antibiotic prescription should be evaluated on a case-by-case basis, rather than as a standard treatment for GI disease. Ultimately the decision of prescribing antibiotics will depend on the severity of the clinical presentation, the results of laboratory testing, and the experience of the clinician.

Tylosin and/or metronidazole are commonly used antibiotics for GI diseases, and have a severe impact on the gut microbiome (97). Antibiotic administration can induce gut dysbiosis, with broad-spectrum antibiotics causing rapid and significant drops in taxonomic richness, diversity, and evenness (97). Once antibiotic treatment is interrupted many bacterial species recover, however, the return to the initial composition is rarely fully achieved (98, 99).

Because of these well-known consequences of antibiotic usage, a renewed interest has been put on probiotics, prebiotics, and synbiotics. While prebiotics are non-digestible food substances, like fiber, that foster the expansion of beneficial bacteria already residing in the host, probiotics supply an exogenous source of live bacteria to the host (100). Synbiotics are products that contain a combination of both. Many different formulations are commercially available, but there is not enough scientific evidence to support one formulation against the others (101).

In dogs, different fibers have been studied for their prebiotic properties, and induce specific changes in the microbiome. Beet pulp (9) was found to increase overall phylum Firmicutes, with increased abundance of class Clostridia and decreased Erysipelotrichi, and decrease phylum Fusobacteria. Potato fiber (102) and soybean husk (103) act mostly by enriching fiber-fermenting Firmicutes bacterial groups, including *Clostridium* clusters IV (e.g., family *Ruminococcaceae, Faecalibacterium prausnitzii*), and XIVa (e.g., family *Lachnospiraceae, Blautia* spp.). Inulin-type fructans also increased Firmicutes but from families *Erysipelotrichaceae* and *Turicibacteraceae* (21). Potato fiber, soybean husk and inulin-type fructans were also found to increase SCFA, including acetate, butyrate and propionate. In addition, inulin-type fructans (21) increased total fecal bile acids, and decreased Proteobacteria (e.g., *Enterobacteriaceae*). Inulin and yeast cell wall were both tested in combination with a raw meat diet (104), and inulin was found to decrease *Enterobacteriaceae* and increase genera *Megamonas* and *Lactobacillus*. Yeast cell wall, instead, lead to an increase in genus *Bifidobacterium*.

Probiotic bacteria are typically not able to colonize the gut due to the competition with the already established microbiota. In a study with healthy dogs (15), the increase in abundance of *Enterococcus* spp. and *Streptococcus* spp. induced by the administration of a synbiotic containing seven probiotic species was only transient and returned to baseline abundance once the treatment was discontinued. Another study found only a small increase in species diversity with the administration of a symbiotic containing *Enterococcus faecium* (105).

However, probiotics can still have beneficial effects through the production of metabolites and antimicrobial peptides that modify the local microbiota and interact with the host immune system (101). In a double-blind placebo-controlled study (106), a sour-milk product containing three canine-derived *Lactobacillus* spp. species was used to treat dogs with AD. The administration of sour-milk based product accelerated normalization of stool consistency and reduced the abundance of an α-toxin-producing *Clostridium perfringens* strain, and *Enterococcus faecium*, which are both potential enteropathogens. In addition, the treatment also increased the well-being of the dogs by maintaining appetite. In sled dogs, who commonly suffer from diarrhea during periods of strenuous exercise, a symbiotic containing three probiotic species that led to a significant rise in fecal *Lactobacillaceae* after 2 weeks of treatment, and had a protective effect during an outbreak of contagious diarrhea despite having no significant impact on overall SCFA production (107).

In dogs with IBD, probiotics are sometimes recommended in combination with standard immunosuppressive treatment. In a study (108), dogs with IBD were randomized to receive standard therapy with or without probiotic. Both treatments modulated the number of mucosal bacteria of IBD dogs in a similar fashion, with increased numbers of bacteria in adherent mucus, and were associated with rapid clinical remission despite no decrease of histopathologic inflammation. Interestingly, though, only dogs receiving probiotic had increased tight junction protein expression, suggesting that, despite the lack of colonization, probiotics may have beneficial effects on mucosal homeostasis.

In another study (109), a multi-strain probiotic was a successful alternative to treatment with a combination protocol (prednisone and metronidazole) in dogs with IBD for 60 days. Clinical scores in both groups significantly decreased over time, although the main clinical sign disappeared faster in the group receiving standard treatment. However, when the gut microbiome was evaluated by qPCR for specific relevant taxa 30 days after the end of treatment, only the group receiving probiotics showed a recovery in *Faecalibacterium* spp. abundance, a butyrate producing bacteria which was not amongst the probiotic strains. No significant changes were observed for any other bacterial groups in response to treatment.

One interesting development of the study of the gut microbiome is fecal microbiota transplantation (FMT), which consists in administering fecal matter from a healthy donor to the patient, usually endoscopically. In humans, fecal transplants have been used with success in the treatment of recurrent *C. difficile* infections for many years, with the goal of restoring the microbiome to inhibit *C. difficile* colonization. FMT is considered a safer and more effective treatment for recurrent *C. difficile* infections compared to standard antibiotic therapy (110, 111). Treatment trials for other diseases with FMT has been reported, including IBD (112). In humans with IBD, success rates varied from 22 to 60.5% (113). In dogs, case-control studies are lacking and case reports use a range of different techniques, making it difficult to make comparisons or to establish their effectiveness (114).

In one of the few case-control studies in dogs so far, puppies infected with parvovirus treated with FMT had significant reduction in hospitalization time and recovered faster than puppies receiving standard treatment (115). However, when oral FMT was used on puppies during weaning in a research setting, no improvement was seen in fecal scores, and FMT did not prevent weaning–associated diarrhea (116). A study (117) reported good results, albeit transient, in a case series of 16 dogs with IBD, with prolonged remission observed when dogs were maintained on a daily oral dosing of frozen donor stool following FMT. In another study (118), successful recovery of a cat with ulcerative colitis was reported after two rounds of FMT.

While promising, the use of FMT to treat dysbiosis and its associated diseases still requires further research to establish the ideal methodology to be applied to dogs. Factors like donor sample preservation (freezing or additives), route (upper GI endoscopy or colonoscopy) and schedule of administration (single transplant or daily administration of capsules) can significantly affect outcomes, and data from human studies does not necessarily translates to dogs due to anatomic and physiologic differences. Hopefully, future studies will allow researchers to fully gauge the potential and eventual limitations of FMT in the treatment of gastrointestinal diseases.

## Conclusions

In conclusion, the composition of the gut microbiome in dogs is correlated with overall health. The gut microbiome is stable in adult healthy dogs, but age, diet, and many other environmental factors may influence the maintenance of a healthy microbiome. The alterations found in diseased animals however are marked, and when they impact the transcriptome, proteome, or metabolome they are termed dysbiosis. Dysbiosis should always be considered when GI tract pathologies are present. The recovery of the microbiome composition does not necessarily correlate with the clinical recovery, and the long-term consequences of such lingering alterations are so far unknown. The identification of bacterial taxa and bacteria-derived compounds involved in the pathogenesis of acute and chronic GI diseases may aid the development of new diagnostic and therapeutic tools, and should be investigated.

## Author Contributions

RP did the majority of the writing and editing. JS provided significant advice on content and editing as this document evolved to its current form.

### Conflict of Interest

The authors declare that the research was conducted in the absence of any commercial or financial relationships that could be construed as a potential conflict of interest.
